# Training residents in minimally invasive surgery; confirming competence or hoping for the best?

**DOI:** 10.1111/vsu.13850

**Published:** 2022-07-30

**Authors:** Boel A. Fransson

**Affiliations:** ^1^ Department of Veterinary Clinical Sciences, College of Veterinary Medicine Washington State University Pullman Washington USA

## Abstract

**Background:**

Veterinary minimally invasive surgery (MIS) is rapidly developing, and most surgeons are performing MIS in their clinical practice. The technical skills of presented surgical techniques are increasingly complex. Required training of American College of Veterinary Surgeons (ACVS) surgical residents in soft tissue MIS (laparoscopy/thoracoscopy) are limited to traditional apprentice training. Unfortunately, such training has been found insufficient to create competent MIS surgeons.

**Aim of the review:**

This review discusses development of MIS training for Doctor of Medicine (M.D.) residents in context of veterinary applicability and investigates comparative evidence for how to best train veterinary residents in soft tissue MIS.

**Conclusions:**

A structured curriculum, with validated tasks and clear training goals have been found imperative for training success. Such a curriculum includes both didactic sessions and manual skills training, with video tutorials and reading material to inform and motivate the residents.

**Implications of key findings:**

ACVS residents and diplomates may benefit if a MIS curriculum was developed and made available to all training programs.

## 1 INTRODUCTION

Almost 40 years ago, the gynecologist Kurt Semm performed the first laparoscopic appendectomy, much to the disdain of some of the general surgeons of the time. Disgruntled surgeons expressed that “such nonsense does not and will never belong to general surgery.”^1^ Despite the initial aversion, by the 1990s the “laparoscopic revolution” was in full swing, and in 2020 it was estimated that more than 14 million laparoscopic surgeries are performed annually in the United State alone.^2^ Millions of patients have experienced the recovery‐associated benefits of minimally invasive surgery (MIS), which has been proven safe, efficacious and cost effective.^3^ However, at least initially, the traditional surgical training was not well adapted to the challenges of this technology, with an increase of certain surgical complications as a result. Over time the training approach “Dry lab on Saturday, Pig lab on Sunday, and Grandma on Monday” has been found wholly inadequate and associated with lacking patient safety.^4^ In fact, the laparoscopic revolution was the main reason for a complete overhaul of M.D. resident surgical education.^4^

Veterinary MIS surgeons were fortunate to avoid much of the controversy our M.D. counterparts experienced, as we were following in their footsteps. We also did not experience the same “revolution,” as our animal patients are unable to express their desire for MIS. In the 1990s and early 2000s veterinary MIS became more prevalent as a variety of straightforward routine procedures and less technically challenging laparoscopic‐assisted procedures became increasingly popular. As MIS has come of age in veterinary medicine, the technical challenge of presented techniques has significantly increased. Procedures where small imprecisions in technique carries risk for high morbidity, such as cholecystectomy and adrenalectomy, have become established advanced MIS procedures. More recently, procedures of high technical challenge including intracorporeal suturing have been described,^5^, ^6^ and this development is likely to continue. These challenging procedures require a high level of surgical skills, and currently we have no mechanism for determining if a surgeon is competent to perform them or not, other than crossing our fingers and hoping for the best. Unless we in veterinary surgery want to experience patient safety concerns like those of human MIS, we may need to initiate a serious conversation about the skills required to perform these procedures, and how we will train these skills in our future surgeons?

## 2 TRAINING METHODS

It is generally accepted that MIS skills are distinctly different than those of open surgery and require specific training, beyond the traditional “see one, do one, teach one” approach. The skill difference relates both to fundamental psychomotor skills, as well as didactic and technical surgical procedure skills.

MIS surgery compared to open surgery is often described as the difference of eating with chopsticks instead of using fork and knife. Before considering performing high morbidity surgery in live patients, the surgeon better be skilled with those chopsticks!

The basic skills have been the topic for intense research, and the need for training initiated a fundamental shift in surgical education bringing simulation training to the surgery realm.^7^ Several psychomotor skills were recognized as imperative, including ambidexterity, hand‐eye coordination, instrument targeting accuracy, and recognition of cues to provide a sense of depth despite the monocular camera view.^8^, ^9^ Several systematic reviews have tried to elucidate the most effective way of training the basic laparoscopic skills.^10^, ^11^, ^12^, ^13^, ^14^, ^15^ Video box‐training carries the advantage of lower equipment cost, and the use of similar instruments, sutures and other materials as those used in the operating room. The interaction between instruments and materials delivers a tactile sensation known as haptic feedback. However, box‐training is limited by low technology physical models which often cannot simulate complications such as bleeding, nor entire surgical procedures. The biggest drawback of the box training is likely that there is no integrated cognitive didactic component and feedback on the performance is not provided. Effective box training may benefit from the trainee, at least occasionally, being proctored by an experienced surgeon, who can provide individualized feedback. This is a challenging commitment for senior surgeons training residents, especially if that surgeon had not received similar mentorship during their training. However, structured training with video demonstrations and clearly stated performance goals seem to be able to replace expert supervision.^16^ Despite all the limitations with traditional box‐training, systematic reviews have not been able to demonstrate clear inferiority of this type of training compared to the much more technologically advanced, and thus expensive, virtual reality trainers.^17^, ^18^

Virtual reality (VR) training includes both basic skills in low fidelity (not lifelike) simulations, as well as high fidelity simulation of entire surgical procedures. VR can simulate complications such as bleeding, bile leakage and slipping of clips. Importantly, the digital systems can easily be paired with cognitive didactic information, which can demonstrate the motor skills needed, provide context, and improve understanding of the task or procedure. These trainers also provide immediate performance feedback, with comparisons to MIS experts' performance benchmarks. However, these sophisticated training machines still have limitations. They are expensive in purchase and maintenance through software upgrades. Also, less expensive units lack haptic feedback, which has been considered a negative for development of suturing skills.^19^

Currently, none of these training modalities are common in veterinary surgery resident training, despite the clearly demonstrated transferability of gained skills into the operating rooms.^10^, ^20^, ^21^ In a 2020 survey of ACVS small animal residents, only 36% had access to any form of simulation training, and of those only half indicated that use of the training equipment was encouraged by senior surgeons.^22^ This is unfortunate, because relying on traditional apprentice training for MIS skills has been generally accepted as inadequate.^12^, ^23^, ^24^, ^25^, ^26^ One can argue that a mere three‐year ACVS resident training program is not designed to produce competent MIS surgeons. Of the 400 small animal cases required, only five are laparoscopy/thoracoscopy cases. This limited case requirement may indicate that the ACVS training only serves as an introduction to MIS. This notion is however not supported by a survey made among ACVS surgeons in 2010, where 86% of small animal and 99% of large animal diplomates performed MIS in their practice, whether they had received training during residency or not.^27^ This demonstrated wide use of MIS may prompt discussions on whether the residency MIS training needs to be expanded?

## 3 EXPERIENCES IN COMPARATIVE TRAINING PROGRAMS

Surgical educators who have made expensive equipment available for resident MIS training, may quickly become disappointed in the lack of voluntary use of the training opportunity. One frustrated resident program director expressed “I can't get the residents to go to the skills lab … They don't believe in ‘home work’ …”.^7^ Unfortunately, unrestricted access to simulator equipment is not effectively motivating surgical residents, with their high clinical responsibilities and limited free time to train.^11^, ^28^ Furthermore, nonsupervised training may not lead to skill improvement,^29^, ^30^ and self‐directed trainees seem to overestimate their own skill increase.^30^ For these and other reasons, M.D. surgical training programs have struggled to incorporate simulators into their curriculum.^31^, ^32^

It has become apparent that the most important contributor to resident learning is a structured curriculum, with validated tasks and clear training goals.^28^ Such a curriculum includes both didactic sessions and manual skills training, with video tutorials and reading material to put the manual skills acquisition into context and to serve as motivation.^28^

The American College of Surgeons (ACS) and Association of Program Directors in Surgery (APDS) created the National Skills Curriculum to provide residency programs with a standardized curriculum. The curriculum entails both open and laparoscopic surgery and was divided into three phases with phase I focusing on basic skills and tasks, using low‐cost laparoscopic box‐training.^33^ Virtual reality training was considered cost‐prohibitive and was not included.^33^ The manual skills component was based on the fundamentals of laparoscopic surgery (FLS), as being the most validated system.^33^ Phase II was dedicated to 15 advanced procedures and phase III was focusing on team‐based competencies.^7^ The implementation of this curriculum was gradual with 41% of ACS residency programs having implemented the curriculum by 2012.^7^ However, more recently the adoption rate has increased to include the vast majority (77%) of accredited residencies.^34^ Regardless of curriculum implementation, since 2009 the American Board of Surgeons (ABS) require general surgery residents to be FLS certified to become board‐eligible.^35^

Barriers to implementation included lack of faculty and resident protected time away from clinics, cost, lack of personnel, lack of faculty incentives, and lack of administrative buy‐in among others.^7^ Another laparoscopic skills curricula, based on both FLS and virtual reality trainers was implemented in a busy residency program, and resident attendance was a major challenge.^36^ The authors concluded that such skills curricula can only be implemented if dedicated personnel and protected training times are provided.^36^

The lessons learnt by implementing training curricula for ACS residents could be important in development of a veterinary surgery resident MIS curriculum.

## 4 VETERINARY MIS TRAINING

Several components of MIS training are already available for ACVS residents, albeit currently not combined into a comprehensive curriculum. Apprentice training within the traditional ACVS training is likely highly variable between institutions, depending on the faculty expertise and interest. Short courses for basic and advanced MIS procedures have been developed and these also contain simulation training elements. Though these are excellent training opportunities, all institutions may not be able to cover the costs for all residents, and the training is massed rather than distributed over time. This author has developed a task training program which lends itself to independent distributed training. However, institutions may not want to provide the needed equipment and without protected resident training time the training may not have the intended effects. Furthermore, a didactic component is not currently paired with the tasks. All these training elements could however be combined and developed into a comprehensive curriculum, which if adopted by the ACVS would improve and standardize resident MIS training. Desire for such a program has already been expressed.^22^ Twelve required components of a MIS curriculum are presented in Table 1.

**TABLE 1:** Components required for successful surgery resident MIS skills training

Factor	Reason
General	
Competency, not mastery, as training goal	Mastery of MIS requires more intense training, likely similar to the ACVS Fellowship training program.
Curriculum provided to residency programs	For national availability and standardization, and to decrease the workload on individual programs.
Simulation Training	
Distributed practice	Massed practice increases fatigue, limits skills development, and decreases skills retention.
Validated tasks with clear performance goals	Nonvalidated tasks may not provide skills that transfer to the operating room.
Didactic teaching	Video tutorials and surgical procedure demonstrations provide context, breaks down skills into appropriate chunks, and motivates trainees.
Protected trainee and mentor time	Without protected time attendance is very low.
Support staff in skills laboratory	For training organization and monitoring, equipment and material/supplies maintenance.
Adequate funding	For equipment, supplies, and staff.
Training in operating room	
Surgical Component training in live animals	For example, laparoscopic entry lacks a high‐fidelity simulation and needs training in OR.
Basic laparoscopic procedures under faculty supervision	Ovariectomy, cryptorchid orchiectomy, and laparoscopic‐assisted procedures are low morbidity if supervised.
Advanced laparoscopic procedures under faculty supervision	Intracorporeally sutured gastropexy is low morbidity if supervised. Pericardiectomy may also be suitable.
Training in Animal Models	
Advanced procedure training	Cholecystectomy, adrenalectomy, diaphragmatic hernia, and many thoracoscopic procedures are associated with risks for life‐threatening complications precluding training in the operating room.

## 5 COMPETENCY, NOT MASTERY, AS TRAINING GOAL

Development of expertise has been generally accepted to require sustained, deliberate practice distributed over long time.^28^, ^37^, ^38^ Though many manual skills can become autonomous with 50 hours of performance, true expertise has been estimated to require over 10 000 hours of deliberate practice.^39^ Focused practice of that magnitude is not possible within the time frame of an ACVS surgery residency. Fortunately, there are now training options for ACVS surgeons with a desire to reach mastery level in MIS, in the ACVS Fellowship training program. Unfortunately, the Fellowship positions are very few in numbers, with only three individuals receiving Fellow status in small animal MIS, and two more currently in training, since the inception of these programs in 2017. However, if ACVS could produce residents that are truly competent in most basic and advanced procedures within the three‐year program, many benefits would likely be extended to our patients, with wider adoption of MIS procedures and increased patient safety.

## 6 CURRICULUM DEVELOPMENT

A curriculum for MIS training has been considered the main factor for success in residency training.^28^, ^33^ If curriculum development is left up to each individual institution one can expect inconsistencies in both quality and implementation. Curricula development is a major undertaking and requires a concerted effort among current specialists to be truly comprehensive. Professional video tutorials with demonstrations of tasks and surgical procedures are expensive and time‐consuming to produce. Assessments to ensure training outcome needs validation and implementation. It seems unnecessary that individual programs should have to re‐invent such a massive “wheel,” whereas if a national program is developed, all institutions training residents would benefit.

## 7 DISTRIBUTED PRACTICE

There is no question, that distributed rather than massed training is required for appropriate skill development.^28^, ^31^, ^37^, ^40^ Little is known about the optimal spacing of sessions, but 1‐hour weekly sessions has been suggested as best suited for resident training.^28^ Therefore, much of the training needs to be available and performed at the residents' institutions to be effective. Conversely, short courses are not suited to provide residents with manual skills. In particular, the manual skills require practice that is distributed over time, and revisited intermittently to avoid skill decay.^41^

## 8 VALIDATED TASKS WITH CLEAR PERFORMANCE GOALS

Medical simulation training today is a billion‐dollar industry, and its market size is projected to quadruple or more by 2030. Therefore, there are endless products available for training, but they all have in common that they are developed for M.D. and not veterinary training. If the training is intended to truly serve as a bridge between didactic learning and real‐life clinical experience, the training modalities must be validated. When the veterinary community has been ascertained that the training task indeed transfers to our operating rooms, the investment in time and money can be justified. After careful task selection, the trainee needs to be provided with clear performance goals. Without clear goals, the training is at risk of becoming repetitive but without appropriate skill development, and the residents are likely to lose motivation under such circumstances.^28^

## 9 DIDACTIC TEACHING

A comprehensive curriculum requires both manual skills training as well as a didactic component; likely both reading material and video demonstrations of tasks and surgical procedures. For competency, careful selection of the most important and prevalent surgical procedures is necessary. Also, experts would have to select one way of procedure performance, rather than teaching all variations presented in the literature, or the material would become overwhelming. Therefore, the didactic teaching component would have to be a concerted effort among current MIS experts to ensure wide adoption and maintained resident motivation.

## 10 PROTECTED TRAINEE AND MENTOR TIME

Any curriculum introduced in the already very busy resident training is likely to fail unless training time is protected for both trainee and the surgeon mentor. Attendance rates in voluntary programs without protected time have proven very low, despite the expressed desire to train.^42^ To ensure that time indeed is protected, the institutions have to be convinced the curriculum is worthwhile and appropriate in scope. Administrators also have to recognize the value of training, to justify the surgeons' participation. The surest way to ensure protected time would be if such training become mandatory, with a subsequent skills test to demonstrate competency.

## 11 SUPPORT STAFF

A functioning simulation laboratory requires staff for a multitude of reasons; scheduling of sessions, reminding residents of sessions, instruction, monitoring of progress, outcomes assessments, troubleshooting equipment, ordering of supplies and in general ensuring a smooth operation.^7^, ^28^, ^36^ Malfunctioning equipment or lack of required disposables will rapidly decrease motivation to practice.

## 12 ADEQUATE FUNDING

For effective simulation training an institution would have to invest in the training equipment, disposable materials, and staffing. An older study indicated investments varying from $300 to 1,000,000.^31^ Advanced technology is available but with associated high costs. For this reason, the ACS/APDS chose box‐training for the manual skills component, to minimize institutions’ investments while still being highly validated.^33^ Hidden costs entail clinicians' time away from clinics and the associated effects on productivity. However, competency in manual skills training has been reached with an average of 10‐hour training,^43^ which should not have severe effects on the case load.

## 13 SURGICAL COMPONENT TRAINING

Certain basic components of MIS are not available in the form of simulation training, and needs training in the operating room, most notably laparoscopic/thoracoscopic entry. However, if covered by the didactic curriculum, the resident could be required to pretrain prior to operating room experience. Such pretraining paired with supervision by attending surgeons provides a reasonable base for gaining experience on the patient base.

## 14 BASIC LAPAROSCOPIC PROCEDURES

Pretraining with didactic and manual skills prior to supervised performance in the OR, renders residents capable of performing basic laparoscopic procedures. This author's 12‐year experience of simulation training of residents have provided an understanding of the dramatic skill improvement by a limited simulation training program. In particular, the most dramatic effects are seen in residents with average innate skills. Interestingly, residents with apparent average innate skill, tended to eventually out‐perform trainees that went into the training with a high skill level and therefore less of a motivation to practice.^44^

## 15 ADVANCED LAPAROSCOPIC PROCEDURES TRAINABLE IN THE OPERATING ROOM

Select advanced procedures are associated with low morbidity and low risk for serious complications, if supervised by an experienced surgeon. Such procedures could be allowed for resident experiences in the OR. At the author's institution, residents are not allowed a primary role in laparoscopic gastropexies, until the simulation curriculum including suturing is completed and the competency test passed. This has served as important motivation to complete the training. We have also noticed that advanced performance on live patients in immediate conjunction with the training program seemed to add significant motivation.^45^

## 16 ADVANCED PROCEDURE TRAINING IN ANIMAL MODELS

Certain advanced procedures come with risks for life‐threatening complications, and these cannot be allowed for resident experiences on the client‐owned animal patients. However, basic competence in these procedures could be reached by didactic training, manual skills training on the components of the procedures, and by procedure training in live animal models. Fortunately, the latter has been made available more recently by institutions offering short courses designed for ACVS residents. A comprehensive curriculum could thus be designed to include such a course as the last phase of training, serving both a training purpose as well as motivation to complete the curriculum.

In conclusion, veterinary MIS has come of age with high complexity of presented procedures. The time may have come to initiate a conversation on how we can ensure that future ACVS surgeons will be competent performing them. A standardized comprehensive curriculum may need to be implemented to ensure adequate resident training. Valuable experiences have been presented after implementation of similar programs for M.D. surgeons and these could help guiding development of a veterinary MIS curriculum.

## CONFLICT OF INTEREST

Dr Fransson has developed the veterinary assessment of laparoscopic skills (VALS) program, a recharge operated service to veterinarians, which does not extend personal financial gain. Training equipment for the VALS program is manufactured by Limbs and Things Inc., Savannah, GA, without royalties or other reimbursement to the author.
